# 3-Chloro-*N*′-(2-chloro-5-nitro­benzyl­idene)­benzohydrazide

**DOI:** 10.1107/S160053681102157X

**Published:** 2011-06-11

**Authors:** Zhen Zhang

**Affiliations:** a Experimental Center, Linyi University, Linyi Shandong 276005, People’s Republic of China

## Abstract

The title mol­ecule, C_14_H_9_Cl_2_N_3_O_3_, has an *E* configuration with respect to the methyl­idene unit. The dihedral angle between the mean planes of the two benzene rings is 12.3 (3)°. In the crystal, mol­ecules are linked *via* bifurcated N—H⋯(O, N) hydrogen bonds into chains along [001].

## Related literature

For the biological applications of hydrazone compounds, see: Ajani *et al.* (2010 ▶); Avaji *et al.* (2009 ▶); Fan *et al.* (2010 ▶); Rasras *et al.* (2010 ▶). For related hydrazone structures, see: Zhang (2011*a* ▶,*b* ▶); Ahmad *et al.* (2010 ▶); Ban (2010 ▶); Ji & Lu (2010 ▶); Shalash *et al.* (2010 ▶).
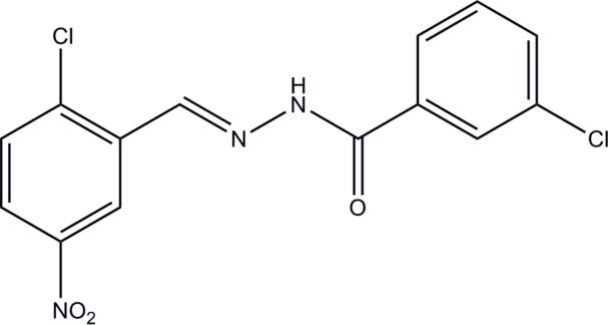


## Experimental

### 

#### Crystal data

C_14_H_9_Cl_2_N_3_O_3_
*M* *_r_* = 338.14
Monoclinic, 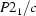

*a* = 7.770 (2) Å
*b* = 24.254 (6) Å
*c* = 7.889 (2) Å
β = 95.383 (3)°
*V* = 1480.1 (7) Å^3^
*Z* = 4
Mo *K*α radiation
μ = 0.45 mm^−1^
*T* = 298 K
0.32 × 0.30 × 0.30 mm

#### Data collection

Bruker SMART APEX CCD diffractometer
Absorption correction: multi-scan (*SADABS*; Bruker, 2009 ▶) *T*_min_ = 0.868, *T*_max_ = 0.876
7538 measured reflections
3016 independent reflections
1607 reflections with *I* > 2σ(*I*)
*R*_int_ = 0.055

#### Refinement

*R*[*F* ^2^ > 2σ(*F* ^2^)] = 0.060
*wR*(*F* ^2^) = 0.152
*S* = 1.04
3016 reflections
202 parameters
1 restraint
H atoms treated by a mixture of independent and constrained refinement
Δρ_max_ = 0.23 e Å^−3^
Δρ_min_ = −0.24 e Å^−3^

### 

Data collection: *APEX2* (Bruker, 2009 ▶); cell refinement: *SAINT* (Bruker, 2009 ▶); data reduction: *SAINT*; program(s) used to solve structure: *SHELXTL* (Sheldrick, 2008 ▶); program(s) used to refine structure: *SHELXTL*; molecular graphics: *SHELXTL*; software used to prepare material for publication: *SHELXTL*.

## Supplementary Material

Crystal structure: contains datablock(s) global, I. DOI: 10.1107/S160053681102157X/lh5264sup1.cif

Structure factors: contains datablock(s) I. DOI: 10.1107/S160053681102157X/lh5264Isup2.hkl

Supplementary material file. DOI: 10.1107/S160053681102157X/lh5264Isup3.cml

Additional supplementary materials:  crystallographic information; 3D view; checkCIF report

## Figures and Tables

**Table 1:** Hydrogen-bond geometry (Å, °)

*D*—H⋯*A*	*D*—H	H⋯*A*	*D*⋯*A*	*D*—H⋯*A*
N2—H2⋯N1^i^	0.89 (1)	2.43 (2)	3.139 (4)	137 (3)
N2—H2⋯O3^i^	0.89 (1)	2.27 (2)	3.095 (4)	154 (3)

Symmetry code: (i) 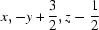.

**Fig. 1.**
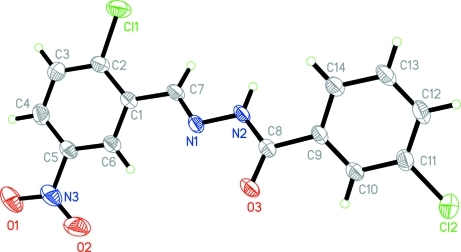
The molecular structure of the title compound, showing displacement ellipsoids at the 30% probability level.

**Fig. 2.**
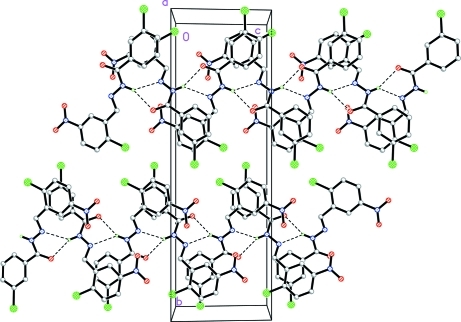
The packing of the title compound. Hydrogen bonds are shown as dashed lines. Only H atoms involved in hydrogen bonds are shown.

## supplementary crystallographic information

### Comment

Hydrazone compounds have received much attention due to their potential applications in biological chemistry (Ajani *et al.*, 2010; Avaji *et al.*, 2009; Fan *et al.*, 2010; Rasras *et al.*, 2010). As a continuation of our work related to hydrazone compounds, the crystal structure of the title compound is reported herein.

The molecule is in a *E* configuration with respect to the methylidene unit (Fig. 1). The bond lengths are comparable to those observed in similar hydrazone compounds (Zhang, 2011*a*,*b*; Ahmad *et al.*, 2010; Ban, 2010; Ji & Lu, 2010; Shalash *et al.*, 2010). The dihedral angle between the mean planes of the two benzene rings is 12.3 (3)°. In the crystal, molecules are linked via bifurcated N—H···(O, N) hydrogen bonds to from chains along [001] (Table 1, Fig. 2).

### Experimental

A methanol solution (50 ml) of 3-chlorobenzohydrazide (0.01 mol) and 2-chloro-5-nitrobenzaldehyde (0.01 mol) was stirred at room temperature for 30 min to give a yellow solution. Yellow block-shaped single crystals suitable for X-ray diffraction were formed by slow evaporation of the solution in air.

### Refinement

The amino atom, H2, was located in a difference Fourier map and refined isotropically, with the N—H distance restrained to 0.90 (1) Å. The remaining H atoms were positioned geometrically and refined using the riding-model approximation, with C—H = 0.93 Å, and with *U*_iso_(H) = 1.2*U*_eq_(C).

### Crystal data

**Table:**

C_14_H_9_Cl_2_N_3_O_3_	*F*(000) = 688
*M**_r_* = 338.14	*D*_x_ = 1.517 Mg m^−^^3^
Monoclinic, *P*2_1_/*c*	Mo *K*α radiation, λ = 0.71073 Å
Hall symbol: -P 2ybc	Cell parameters from 814 reflections
*a* = 7.770 (2) Å	θ = 2.6–24.5°
*b* = 24.254 (6) Å	µ = 0.45 mm^−^^1^
*c* = 7.889 (2) Å	*T* = 298 K
β = 95.383 (3)°	Block, yellow
*V* = 1480.1 (7) Å^3^	0.32 × 0.30 × 0.30 mm
*Z* = 4	

### Data collection

**Table:**

Bruker SMART APEX CCD diffractometer	3016 independent reflections
Radiation source: fine-focus sealed tube	1607 reflections with *I* > 2σ(*I*)
graphite	*R*_int_ = 0.055
ω scans	θ_max_ = 26.5°, θ_min_ = 2.6°
Absorption correction: multi-scan (*SADABS*; Bruker, 2009)	*h* = −5→9
*T*_min_ = 0.868, *T*_max_ = 0.876	*k* = −30→30
7538 measured reflections	*l* = −9→9

### Refinement

**Table:**

Refinement on *F*^2^	Primary atom site location: structure-invariant direct methods
Least-squares matrix: full	Secondary atom site location: difference Fourier map
*R*[*F*^2^ > 2σ(*F*^2^)] = 0.060	Hydrogen site location: inferred from neighbouring sites
*wR*(*F*^2^) = 0.152	H atoms treated by a mixture of independent and constrained refinement
*S* = 1.04	*w* = 1/[σ^2^(*F*_o_^2^) + (0.0443*P*)^2^ + 0.2711*P*] where *P* = (*F*_o_^2^ + 2*F*_c_^2^)/3
3016 reflections	(Δ/σ)_max_ < 0.001
202 parameters	Δρ_max_ = 0.23 e Å^−^^3^
1 restraint	Δρ_min_ = −0.24 e Å^−^^3^

### Special details

**Table:**

Geometry. All e.s.d.'s (except the e.s.d. in the dihedral angle between two l.s. planes) are estimated using the full covariance matrix. The cell e.s.d.'s are taken into account individually in the estimation of e.s.d.'s in distances, angles and torsion angles; correlations between e.s.d.'s in cell parameters are only used when they are defined by crystal symmetry. An approximate (isotropic) treatment of cell e.s.d.'s is used for estimating e.s.d.'s involving l.s. planes.
Refinement. Refinement of *F*^2^ against ALL reflections. The weighted *R*-factor *wR* and goodness of fit *S* are based on *F*^2^, conventional *R*-factors *R* are based on *F*, with *F* set to zero for negative *F*^2^. The threshold expression of *F*^2^ > σ(*F*^2^) is used only for calculating *R*-factors(gt) *etc*. and is not relevant to the choice of reflections for refinement. *R*-factors based on *F*^2^ are statistically about twice as large as those based on *F*, and *R*- factors based on ALL data will be even larger.

### Fractional atomic coordinates and isotropic or equivalent isotropic displacement parameters (Å^2^)

**Table:**

	*x*	*y*	*z*	*U*_iso_*/*U*_eq_	
Cl1	0.33815 (15)	0.55819 (4)	0.48098 (13)	0.0786 (4)	
Cl2	0.02262 (16)	0.99758 (4)	0.26818 (14)	0.0827 (4)	
N1	0.2837 (3)	0.72544 (12)	0.6153 (3)	0.0429 (7)	
N2	0.1950 (4)	0.75727 (12)	0.4918 (3)	0.0459 (7)	
N3	0.6550 (5)	0.65111 (19)	1.1284 (4)	0.0771 (11)	
O1	0.7009 (5)	0.61872 (16)	1.2395 (4)	0.1306 (15)	
O2	0.6670 (5)	0.70061 (16)	1.1441 (4)	0.1060 (12)	
O3	0.1450 (4)	0.82247 (10)	0.6847 (3)	0.0701 (8)	
C1	0.4302 (4)	0.64304 (14)	0.6916 (4)	0.0425 (8)	
C2	0.4361 (4)	0.58649 (14)	0.6668 (4)	0.0493 (9)	
C3	0.5135 (5)	0.55147 (15)	0.7884 (5)	0.0591 (10)	
H3	0.5166	0.5137	0.7681	0.071*	
C4	0.5859 (5)	0.57270 (16)	0.9396 (5)	0.0602 (10)	
H4	0.6375	0.5495	1.0235	0.072*	
C5	0.5812 (4)	0.62850 (16)	0.9653 (4)	0.0513 (9)	
C6	0.5069 (4)	0.66400 (14)	0.8457 (4)	0.0464 (9)	
H6	0.5073	0.7017	0.8665	0.056*	
C7	0.3405 (4)	0.67978 (15)	0.5662 (4)	0.0469 (9)	
H7	0.3252	0.6698	0.4519	0.056*	
C8	0.1301 (5)	0.80617 (14)	0.5386 (4)	0.0467 (9)	
C9	0.0426 (4)	0.83855 (13)	0.3958 (4)	0.0427 (8)	
C10	0.0624 (4)	0.89538 (14)	0.4003 (4)	0.0475 (9)	
H10	0.1224	0.9123	0.4938	0.057*	
C11	−0.0076 (5)	0.92655 (15)	0.2651 (4)	0.0520 (9)	
C12	−0.1003 (5)	0.90243 (17)	0.1273 (5)	0.0587 (10)	
H12	−0.1459	0.9239	0.0362	0.070*	
C13	−0.1246 (5)	0.84646 (16)	0.1259 (4)	0.0574 (10)	
H13	−0.1891	0.8300	0.0342	0.069*	
C14	−0.0544 (4)	0.81411 (15)	0.2593 (4)	0.0472 (9)	
H14	−0.0720	0.7762	0.2576	0.057*	
H2	0.193 (4)	0.7439 (12)	0.386 (2)	0.057*	

### Atomic displacement parameters (Å^2^)

**Table:**

	*U*^11^	*U*^22^	*U*^33^	*U*^12^	*U*^13^	*U*^23^
Cl1	0.0933 (9)	0.0746 (7)	0.0636 (7)	0.0081 (6)	−0.0147 (6)	−0.0264 (6)
Cl2	0.1195 (10)	0.0564 (7)	0.0692 (7)	0.0114 (6)	−0.0070 (7)	0.0126 (5)
N1	0.0476 (17)	0.0491 (17)	0.0306 (15)	0.0043 (14)	−0.0040 (13)	0.0048 (13)
N2	0.0595 (19)	0.0541 (19)	0.0229 (14)	0.0092 (15)	−0.0032 (13)	0.0008 (13)
N3	0.084 (3)	0.092 (3)	0.050 (2)	0.005 (2)	−0.0218 (19)	0.002 (2)
O1	0.195 (4)	0.126 (3)	0.058 (2)	0.012 (3)	−0.058 (2)	0.018 (2)
O2	0.134 (3)	0.094 (3)	0.080 (2)	0.007 (2)	−0.044 (2)	−0.024 (2)
O3	0.114 (2)	0.0647 (17)	0.0285 (13)	0.0229 (16)	−0.0129 (14)	−0.0066 (12)
C1	0.041 (2)	0.051 (2)	0.0342 (18)	0.0037 (16)	−0.0001 (15)	−0.0010 (15)
C2	0.051 (2)	0.054 (2)	0.041 (2)	0.0050 (18)	−0.0016 (17)	−0.0074 (17)
C3	0.070 (3)	0.047 (2)	0.060 (3)	0.0069 (19)	0.001 (2)	0.0013 (18)
C4	0.066 (3)	0.061 (3)	0.052 (2)	0.008 (2)	−0.003 (2)	0.013 (2)
C5	0.048 (2)	0.064 (3)	0.039 (2)	0.0007 (18)	−0.0092 (17)	0.0020 (18)
C6	0.046 (2)	0.052 (2)	0.040 (2)	0.0069 (17)	−0.0018 (17)	−0.0033 (16)
C7	0.054 (2)	0.056 (2)	0.0293 (18)	0.0050 (19)	−0.0023 (16)	−0.0036 (16)
C8	0.056 (2)	0.049 (2)	0.034 (2)	0.0029 (18)	−0.0014 (17)	0.0023 (16)
C9	0.047 (2)	0.052 (2)	0.0289 (18)	0.0104 (17)	−0.0008 (15)	0.0027 (15)
C10	0.058 (2)	0.054 (2)	0.0304 (19)	0.0052 (18)	−0.0012 (16)	−0.0006 (16)
C11	0.060 (2)	0.055 (2)	0.040 (2)	0.0103 (19)	0.0020 (18)	0.0079 (17)
C12	0.060 (3)	0.074 (3)	0.040 (2)	0.018 (2)	−0.0058 (19)	0.0102 (19)
C13	0.061 (2)	0.074 (3)	0.034 (2)	0.010 (2)	−0.0105 (18)	−0.0010 (19)
C14	0.049 (2)	0.055 (2)	0.0365 (19)	0.0067 (17)	0.0002 (17)	−0.0011 (16)

### Geometric parameters (Å, °)

**Table:**

Cl1—C2	1.729 (3)	C4—C5	1.370 (5)
Cl2—C11	1.738 (4)	C4—H4	0.9300
N1—C7	1.266 (4)	C5—C6	1.365 (4)
N1—N2	1.376 (3)	C6—H6	0.9300
N2—C8	1.354 (4)	C7—H7	0.9300
N2—H2	0.893 (10)	C8—C9	1.485 (4)
N3—O1	1.206 (4)	C9—C10	1.387 (4)
N3—O2	1.210 (4)	C9—C14	1.388 (4)
N3—C5	1.465 (5)	C10—C11	1.377 (4)
O3—C8	1.214 (4)	C10—H10	0.9300
C1—C2	1.387 (5)	C11—C12	1.377 (5)
C1—C6	1.398 (4)	C12—C13	1.371 (5)
C1—C7	1.460 (4)	C12—H12	0.9300
C2—C3	1.377 (5)	C13—C14	1.383 (4)
C3—C4	1.371 (5)	C13—H13	0.9300
C3—H3	0.9300	C14—H14	0.9300
			
C7—N1—N2	116.1 (3)	N1—C7—C1	119.0 (3)
C8—N2—N1	118.2 (3)	N1—C7—H7	120.5
C8—N2—H2	127 (2)	C1—C7—H7	120.5
N1—N2—H2	115 (2)	O3—C8—N2	122.7 (3)
O1—N3—O2	123.9 (4)	O3—C8—C9	123.0 (3)
O1—N3—C5	117.3 (4)	N2—C8—C9	114.3 (3)
O2—N3—C5	118.8 (3)	C10—C9—C14	119.7 (3)
C2—C1—C6	117.7 (3)	C10—C9—C8	117.7 (3)
C2—C1—C7	121.9 (3)	C14—C9—C8	122.6 (3)
C6—C1—C7	120.3 (3)	C11—C10—C9	119.4 (3)
C3—C2—C1	122.0 (3)	C11—C10—H10	120.3
C3—C2—Cl1	118.3 (3)	C9—C10—H10	120.3
C1—C2—Cl1	119.7 (3)	C12—C11—C10	121.2 (4)
C4—C3—C2	119.4 (3)	C12—C11—Cl2	119.5 (3)
C4—C3—H3	120.3	C10—C11—Cl2	119.4 (3)
C2—C3—H3	120.3	C13—C12—C11	119.3 (3)
C5—C4—C3	119.1 (3)	C13—C12—H12	120.4
C5—C4—H4	120.5	C11—C12—H12	120.4
C3—C4—H4	120.5	C12—C13—C14	120.8 (3)
C6—C5—C4	122.4 (3)	C12—C13—H13	119.6
C6—C5—N3	118.5 (4)	C14—C13—H13	119.6
C4—C5—N3	119.1 (3)	C13—C14—C9	119.7 (3)
C5—C6—C1	119.3 (3)	C13—C14—H14	120.2
C5—C6—H6	120.3	C9—C14—H14	120.2
C1—C6—H6	120.3		

### Hydrogen-bond geometry (Å, °)

**Table:**

*D*—H···*A*	*D*—H	H···*A*	*D*···*A*	*D*—H···*A*
N2—H2···N1^i^	0.89 (1)	2.43 (2)	3.139 (4)	137 (3)
N2—H2···O3^i^	0.89 (1)	2.27 (2)	3.095 (4)	154 (3)

Symmetry codes: (i) *x*, −*y*+3/2, *z*−1/2.
